# Oncocytoma of the Deep Lobe of the Parotid Gland

**DOI:** 10.3889/oamjms.2016.048

**Published:** 2016-03-23

**Authors:** Vladimir Popovski, Alberto Benedetti, Danica Popovik Monevska, Aleksandar Grcev, Predrag Serafimovski, Ruse Pecanovski, Aleksandar Stamatoski

**Affiliations:** 1 *University Clinic for Maxillofacial Surgery, Faculty of Dental Medicine, Ss Cyril and Methodius University of Skopje, Skopje, Republic of Macedonia*; 2 *Faculty of Dental Medicine, Ss Cyril and Methodius University of Skopje, Skopje, Republic of Macedonia*

**Keywords:** Salivary gland tumors, Parotid gland neoplasm, Oncocytoma, MRI, Parotidectomy

## Abstract

**BACKGROUND:**

Oncocytoma or oxyphilic adenoma is uncommon salivary gland tumour, occurs predominantly in the in patients older than 60 years of age. Clinically oncocytoma resemble other salivary tumours while histology is typically consisting of oncocytes with many hyperplastic mitochondria. It usually occurs in the parotid gland. Because the features of oncocytoma are similar to those of other benign and low-grade malignant salivary tumours, clinical diagnosis is often challenging.

**CASE PRESENTATION:**

This report presents the pathologic and imaging findings of an oncocytoma arising in the deep lobe of the right parotid gland in a 74-year-old male. Oncocytoma was diagnosed on the basis of histological, magnetic resonance imaging (MRI), scintigraphic findings and fine needle aspiration cytology (FNAC).

**CONCLUSION:**

This case was unique because in the literature there are few articles about the rare presentation and deep lobe location of this type of parotid oncocytoma.

## Introduction

Oncocytoma or oxyphilic adenoma is a rare benign salivary gland tumor, which mostly occurs in the parotid glands, few affect the submandibular and rarely affect the minor salivary glands [1-4]. Oncocytoma of parotid gland occurs most commonly in patients older than 60 years of age. Oncocytomas are composed of mitochondria-rich epithelial cells called oncocytes [2, 5]. The oncocytes may be present of conditions ranging from hyperplasia to malignant lesions, but the presence of true oncocytes within the entire lesion is a hallmark of oncocytomas [6, 9]. Histologically there are three distinct types: oncocytosis, oncocytoma (the most common form) and oncocytic carcinoma [6].

The clinical presentation of oncocytomas is usually identical to other benign salivary tumors. The image modalities of choice to identify the well-defined homogeneous mass are CT Scan MRI, ultrasonography, radionuclide salivary scintigraphy and positron emission tomography CT scans [11, 12].

Complete surgical removal with superficial or total parotidectomy is the treatment of choice, whereas radiation therapy is not indicated because oncocytes are radioresistant [8]. Recurrence is unusual and probably results from incomplete initial resection. Malignant transformation is occasionally reported [13].

Long-term follow-up using imaging refinements is recommended to evaluate the patient’s progress. Here we are presenting the case of a 74-years-old male patient with right oncocytoma of the deep lobe of the parotid gland extending into para pharyngeal space.

## Case Report

**Figure 1:**
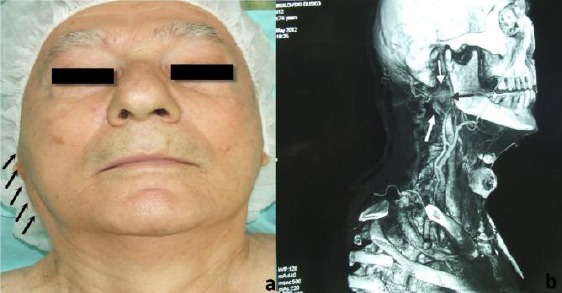
*a) Oncocytoma of the right parotid gland; b) MRI of the oncocytoma in deep lobe of the parotid gland*.

A 74-year-old man underwent a computed tomographic (CT) scan to determine the status of cervical lymph node involvement and discrete swelling of the right parotid gland. One year before, the patient had undergone partial resection of soft palate for mucoepidermoid carcinoma. Neck lymph node involvement was negative, but 32 x 24 mm well-circumscribed mass was incidentally detected in the deep lobe of the right parotid gland [Fig. 1a]. MRI of oropharyngeal and neck region was performed. The MR images showed a well-circumscribed mass in the deep lobe of the right parotid gland with extension to para pharyngeal space [Fig. 1b].

**Figure 2:**
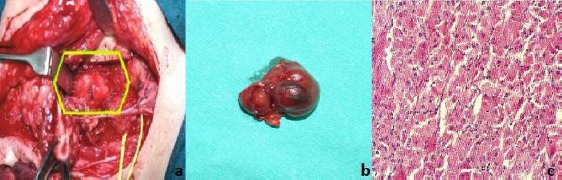
*a) Operative approach for removal of oncocytoma in para pharyngeal space; b) Macroscopic appearance of the tumour mass; c) Oxyphil adenoma - oncocytoma, delicate and vascular stroma oncocytic cells (H&E x 450)*.

The mass showed low-signal intensity to parotid gland parenchyma on T1- and T2-weighted images and homogeneous enhancement after intravenous gadolinium-based contrast medium administration. On the basis of these findings, the lesion was diagnosed as a deep lobe parotid tumour. FNAC with screening performance was applied immediately prior to operative treatment. A parotidectomy was performed with mandibular osteotomy, selectively excising a portion of a deep lobe of the right parotid gland [Fig. 2a]. Postoperatively, an operative specimen with the tumour was examined macroscopically and histologically. Tumour was 32 x 24 mm in size, was resilient, soft, and surrounded by a capsule. It did not adhere to the surrounding tissues. The surface of the tumour was soft, homogeneous, and yellowish brown [Fig. 2b]. Microscopic examination showed large cells separated by exceedingly obscure stroma [Fig. 2c]. The cytoplasm was bright eosinophilic with eosinophilic granules. The cells contained relatively small, round, vesicular nuclei with no or more nucleoli. Mitotic figures were absent. There was a scattering of ducts, suggesting remnants of a duct system. The final histological diagnosis was an oncocytoma. No capsule is discernible in microscopic features. PTAH stain was done to confirm that the eosinophilic granules were mitochondria.

Postoperative period was uneventful. The patient was treated with antibiotics and analgesics for five days. There was no clinical evidence of neurological deficit and was discharged with good health on the third day after surgery. The patient was comfortable at review after two months.

## Discussion

Oncocytoma accounts for less than 1 - 2.2 % of all salivary gland tumours [3, 8-10]. This tumour is composed of oncocytes, transformed cells originating from normal salivary tissue that represents parenchymal elements of acini or intratubular ducts. Oncocytic mitochondria gland produces minimal amounts of adenosine triphosphate [7, 8]. Oncocyte compensates this defect by an increase in numbers of mitochondria, a hallmark of this tumour. Cells with oncocytic features may also be seen in tumors such as pleomorphic adenoma, mucoepidermoid carcinoma, rarely acinic cell tumors, salivary duct carcinoma and the uncommon oncocytic papillary cystadenoma.

The parotid gland is a preferred site of oncocytoma. The tumours are generally noncystic, encapsulated, and rarely exceed 5.0 cm in diameter. Their location at onset is usually unilateral, but sometimes multiple or bilateral (7%) oncocytomas are seen [1, 8, 11, 12]. The aetiology of oncocytoma is unclear because of rarity and variable characteristics of this tumour and most of them are of benign nature. Most patients are in their sixth to eight decades, with no sex predilection [5-8].

The growth grade rate is typically slow. Although malignant transformation and local recurrence of this tumour are unusual and uncommon, clinical follow-up is important because malignant oncocytoma may not be correctly diagnosed owing to histological similarities with benign oncocytoma [3]. Diagnostic evaluation of tumours located in the deep lobe of parotid gland can be challenging, but histopathology remains the gold standard method for a final decision. CT and conventional MRI are presently image modalities of choice used especially in the evaluation of para-pharyngeal space lesions [8, 9]. The important differential diagnosis includes the Warthin’s tumour and basal cell adenomas [3, 7, 8]

Chakrabarti [2] reported that diagnosis of oncocytic lesion of parotid gland by FNAC pose a diagnostic dilemma for histopathologist.

We have evaluated the present case with scintigraphy and supplementary MRI. Adenolymphoma -Warthin’s tumour and oncocytomas reveal intense uptake with marked retention of activity after stimulation on salivary radionuclide imaging while other primary or metastatic malignancies appear cold on radionuclide scintigraphy with technetium-99m-pertechneate. In general, MR images of Warthin’s tumours show decreased signal intensity on T1-weighted images and increased signal intensity on T2-weighted images.

In our patient, oncocytoma showed decreased signal intensity on both T1- and T2-weighted images. This difference made it possible to distinguish oncocytoma from Warthin’s tumour. The decreased signal intensity on both T1- and T2-weighted images is attributed to the high cellularity and low free water content of oncocytoma.

Fine-needle aspiration cytology (FNAC) is another fairly accurate preoperative procedure for the diagnosis of parotid tumours. Capone et al., [5] reported sensitivity in the detection of oncocytic neoplasms of the parotid gland by FNAC of 29%.

Because the scintigraphic findings of the present case were not clearly compatible with Warthin’s tumours or oncocytoma, preoperative FNAC analysis under ultrasonography guidance was performed. The postoperative histological examination was performed for final evaluation of the tumour.

In conclusion, oncocytoma of the deep lobe of the parotid gland is extremely rare but have distinctive imaging characteristics that have not been described for other benign or malignant parotid gland tumors. The other salivary gland tumors where oncocytes are found can usually be differentiated on the basis of their more typical cytologic details.

Imaging findings are helpful in preoperative assessment to distinguish these benign lesions from other parotid gland tumors in different clinical circumstances. Through this case, we highlight the importance of histopathological examination in the positive diagnosis of parotid oncocytoma as well as in its differential diagnosis and also the importance of FNAC.
